# P7C3 Inhibits LPS-Induced Microglial Activation to Protect Dopaminergic Neurons Against Inflammatory Factor-Induced Cell Death *in vitro* and *in vivo*

**DOI:** 10.3389/fncel.2018.00400

**Published:** 2018-11-05

**Authors:** Chao Gu, Qingsong Hu, Jiayuan Wu, Chenchen Mu, Haigang Ren, Chun-Feng Liu, Guanghui Wang

**Affiliations:** ^1^Laboratory of Molecular Neuropathology, Jiangsu Key laboratory of Translational Research and Therapy for Neuropsychiatric Disorders & Department of Pharmacology, College of Pharmaceutical Sciences, Soochow University, Suzhou, China; ^2^The Key Laboratory, The Second Affiliated Hospital of Jiaxing University, Hangzhou, China; ^3^Institute of Neuroscience, Soochow University, Suzhou, China; ^4^Department of Neurology and Suzhou Clinical Research Center of Neurological Disease, The Second Affiliated Hospital of Soochow University, Suzhou, China

**Keywords:** P7C3, lipopolysaccharide (LPS), microglia, neuroinflammation, Parkinson’s disease (PD), nuclear factor κB (NF-κB), IκB kinase (IKK)

## Abstract

Parkinson’s disease (PD) is the second most common neurodegenerative disorder. Although its pathogenesis remains unclear, growing evidencce suggests that microglia-mediated neuroinflammation contributes greatly to the progression of PD. P7C3, an aminopropyl carbazole, possesses significant neuroprotective effects in several neurodegenerative disease animal models, including PD. In this study, we designed to investigate the effects of P7C3 on neuroinflammation. We showed that P7C3 specially suppressed the expression of lipopolysaccharide (LPS)-induced pro-inflammatory factors but not influenced the anti-inflammatory factors in microglia. The inhibition of the nuclear factor κB (NF-κB) signaling pathway was involved in the mechanisms of the anti-inflammatory effects by P7C3. LPS-induced activation of IκB kinase (IKK), degradation of the inhibitory κB alpha (IκBα) and nuclear translocation of NF-κB can be attenuated by the pretreatment of P7C3 in microglia. Furthermore, in LPS-treated microglia, P7C3-pretreatment decreased the toxicity of conditioned media to MES23.5 cells (a dopaminergic (DA) cell line). Most importantly, the anti-inflammatory effects of P7C3 were observed in LPS-stimulated mouse model. In general, our study demonstrates that P7C3 inhibits LPS-induced microglial activation through repressing the NF-κB pathway both *in vivo* and *in vitro*, providing a theoretical basis for P7C3 in anti-inflammation.

## Introduction

Parkinson’s disease (PD) is one of the most prevalent neurodegenerative diseases, ranking only second to Alzheimer’s disease (Samii et al., 2004). Its most typical pathological feature encompasses the preferential loss of dopaminergic (DA) neurons in the substantia nigra pars compacta (SNpc), resulting in dopamine depletion in the striatum (Thomas and Beal, 2007). Loss of DA neurons is able to cause the early cardinal clinical symptoms including bradykinesia, rest tremor, rigidity and damage of postural reflexes. More advanced stages of PD are connected with behavioral and cognitive obstacles (Jankovic, 2008). Although the pathological causes of the DA neuronal death are not completely understood, a mass of evidence clarifies that mitochondrial dysfunction and oxidative stress are deemed to be common elements in the pathological process of PD (Subramaniam and Chesselet, 2013).

Interestingly, a growing body of evidence suggests that microglia-mediated neuroinfammation is involved in the DA neuronal degeneration (Block et al., 2007; Kempuraj et al., 2016). Many inflammatory factors, like interleukin-1β (IL-1β) and tumor necrosis factor-α (TNF-α), are detected in postmortem PD brains (Sawada et al., 2006). Amounts of studies have suggested that neuroinflammation plays an essential role in the etiology of PD (Glass et al., 2010; Yan et al., 2014). Toll like receptor 4 (TLR4) is an important Pattern Recognition Receptor (PRR) expressed in microglia and is responsible for microglial activation (Liu et al., 2012). Lipopolysaccharide (LPS), an exogenous ligand of TLR4 and an activator of microglia, is capable of inducing microglia-mediated neuroinflammation (Monje et al., 2003), which can further lead to the DA neuronal death in the SNpc (Dutta et al., 2008). An epidemiologic study shows that taking non-steroidal anti-inflammatory drugs might be able to decrease the risk of developing PD (Moore et al., 2010), suggesting that anti-inflammation might be a potential therapeutic strategy of PD (Bassani et al., 2015). Therefore, neuroinflammation is closely associated with the pathogenesis of PD, meanwhile, new therapies or drugs targeting neuroinflammation are in urgent need of mitigating the develepment of PD.

P7C3, an aminopropyl carbazole small compound, was first introduced to possess significant neuroprotective ability in newborn hippocampal neurons of the dentate gyrus (Pieper et al., 2010). Thereafter, P7C3 was identified to exert potent protective efficacy in several animal models of nerve-related diseases, including traumatic brain injury (Yin et al., 2014), retinal degeneration (Asai-Coakwell et al., 2013), depressive disorder (Walker et al., 2015), peripheral nerve degeneration (Kemp et al., 2015) and amyotrophic lateral sclerosis (Tesla et al., 2012). Most importantly, P7C3 possesses remarkable neuroprotection efficacy in several neurodegenerative diseases, including PD (De Jesús-Cortés et al., 2012). P7C3 obviously decreases the tyrosine hydroxylase (TH) neuronal loss in the SNpc and striatum in MPTP (De Jesús-Cortés et al., 2012; Gu et al., 2017) mouse or 6-hydroxydopamine (6-OHDA; De Jesús-Cortés et al., 2015) rat model of PD. Since the potent neuroprotection of P7C3 in animal models of PD involving multiple pathways, we wonder whether P7C3 has any influence on inflammation. Up to date, the role of P7C3 in microglia-mediated neuroinflammation is completely unknown.

In the present study, we demonstrated that P7C3 showed great potency of inhibiting the LPS-induced microglial activation both *in vivo* and *in vitro*, relying on its ability of repressing the NF-κB activation induced by LPS. Furthermore, P7C3 suppressed the microglia-mediated neurotoxicity both *in vivo* and *in vitro*.

## Materials and Methods

### Animal Experiments

Male C57BL/6 mice, 25–30 g, 6–8 weeks, were purchased from SLACCAL Lab Animal Ltd (Shanghai, China). The mice were housed under conditions at 20°C–26°C, 50%–60% relative humidity and a 12 h light and a 12 h dark cycle. The animals were raised with free food and water. For total RNA extraction, mice midbrians were homogenized in lysis buffer, and total RNA was isolated with a protein and RNA extraction kit (Takara, Otsu, Shiga, Japan) according to the manufacturer’s recommendations. All experiments were approved and conducted according to the Regulations of Experimental Animal Administration issued by the Animal Committee of Soochow University. For drug treatment *in vivo*, the mice were divided into four groups randomly: (1) vehicle + PBS group; (2) P7C3 + PBS group; (3) vehicle + LPS group; and (4) P7C3 + LPS group. Each mouse in the group (2) and (4) were treated twice daily with P7C3 at a dose of 20 mg/kg/d for consecutive 21 days through intraperitoneal injection, while each mouse in the other groups received an equal volume of vehicle. On the day 22, the mice in the group (3) and (4) were delivered with LPS (1 mg/ml) to the bilateral SN by stereotaxic injection at AP injection at AP −3.3 mm, ML ±1.2 mm, DV −4.6 mm from Bregma.

### Cell Culture and Drug Treatment

BV2 cells, a murine microglial cell line, were maintained in Dulbecco’s modified Eagle’s medium (DMEM) with 10% heat-inactivated fetal bovine serum, 100 μg/mL penicillin and 100 μg/mL streptomycin. MES23.5 cells, a DA cell line, were cultured in DMEM/F12 with Sato’s components containing 5% heat-inactivated fetal bovine serum including penicillin (100 mg/ml) and streptomycin (100 mg/ml; Gu et al., 2017). Primary cultured microglia were obtained and cultured as described previuosly (Xia et al., 2015). P7C3 was purchased from Hanxiang technology (Shanghai, China) and dissolved in dimethyl sulfoxide (DMSO). For *in vivo* experiments, P7C3 was firstly prepared as a stock in DMSO at a concentration of 50 mg/ml. The compound was diluted to a final formulation of 3% DMSO/10% cremophor EL (Sigma, St. Louis, MO, USA)/87.5% D5W (5% dextrose in water, pH 7.2). Adult mice were dosed IP in a total volume of 0.2 ml. LPS was purchased from Sigma (St. Louis, MO, USA) and dissolved in PBS. For detection of phosphorylation of IκB kinase (IKK) and the degradation of inhibitory κB alpha (IκBα), BV2 cells were pretreated with P7C3 for 2 h, and then exposed to LPS for 10 min. To explore the nuclear translocation of NF-κB p65 with subcellular fractionation assay or immunofluorescent staining, BV2 cells were pretreated with P7C3 for 2 h, and were then exposed to LPS for 15 min. For determing mRNA expression and the release of pro-inflammatory factors with RT-PCR and ELISA analyses, BV2 cells were pretreated with P7C3 for 2 h, and were then incubated with LPS for 6 h or 24 h. To investigate the protein levels of iNOS and COX-2, BV2 cells were pretreated with P7C3 for 2 h, followed by a treatment of LPS for 24 h.

### Cell Viability Assay

Cell viability was detected with a MTT assay. BV2 cells were administrated with varying concentrations of P7C3 with or without the existence of LPS. After 24 h treatment, BV2 cells were incubated with 0.5 mg/mL 3-(4, 5-dimethylthiazol-2-yl)-2, 5-diphenyltetrazolium bromide (MTT) at 37°C for 2 h. Then, this reaction was stopped with 150 μL of dimethylsulfoxide (DMSO). The absorbance was assessed at 570 nm to determine cell viability.

### RNA Extraction, Reverse Transcription-PCR and Real-Time Quantitative RT-PCR

BV2 cells were treated with varying concentrations of P7C3 with or without LPS. The total RNA was isolated with TRIzol Reagent (Invitrogen, Carlsbad, CA, USA). Then, the cDNA was obtained using PrimeScript RT Master Mix (Takara, Otsu, Shiga, Japan). Subsequently, real-time quantitative PCR was performed using SYBR Green PCR Master Mix (Applied Biosystems, Warrington, Cheshire, UK) and the products were measured using an Applied Biosystems 7500 Real Time PCR System (Applied Biosystems, Warrington, Cheshire, UK). The sequences of PCR primers were as follows: mouse *β-actin*: 5′-GACCTGACTGACTACCTC-3′ and 5′-GACAGCGAGGCCAGGATG-3′, mouse *iNOS*: 5′-TCCCAGCCTGCCCCTTCAAT-3′ and 5′-CGGATCTCTCTCCTCCTGGG-3′, mouse *COX-2*: 5′-CAGGCTGAACTTCGAAACA-3′ and 5′-GCTCACGAGGCCACTGATACCTA-3′, mouse *TNF-α*: 5′-CATCTTCTCAAAATTCGAGTGACAA-3′ and 5′-TGGGAGTAGACAAGGTACAACCC-3′, mouse *IL-6*: 5′-GCTATGAAGTTCCTCTCTGC-3′ and 5′-CTAGGTTTGCCGAGTAGATC-3′, mouse *Arginase1*: 5′-TTAGGCCAAGGTGCTTGCTGCC-3′ and 5′-TACCATGGCCCTGAGGAGGTTC-3′, mouse *IL-10*: 5′-GGCAGAGAACCATGGCCCAGAA-3′ and 5′-AATCGATGACAGCGCCTCAGCC-3′, mouse *CD206*: 5′-TCAGCTATTGGACGCGAGGCA-3′ and 5′-TCCGGGTTGCAAGTTGCCGT-3′. Relative mRNA expression level was calculated by the 2^ΔΔCT^ method.

### Immunoblot Analysis and Antibodies

Cells were lysed in 1× SDS lysis buffer (150 mmol/L NaCl, 25 mmol/L Tris-HCl, pH 7.6, 1% sodium deoxycholate, and 1% NP-40) in the presence of a protease inhibitor cocktail (Roche). Nearly 20 μg of cell lysate was isolated with SDS-PAGE and then transferred onto a PVDF membrane (Millipore, Billerica, MA, USA). The PVDF menbranes were then blocked with 10% skimmed milk for 30 min. Immunoblot analysis was performed with the following primary antibodies: anti-α-Tubulin, anti-COX-2, anti-Histone 2B, anti-IκBα, anti-iNOS and anti-p-p65 antibodies (Abcam, Cambridge, UK); anti-IKK and anti-p-IKK antibodies (Cell Signaling Technology, Danvers, MA, USA); anti-p65 antibodies (Santa Cruz Biotechnology, Santa Cruz, CA, USA); anti-GAPDH antibodies (Millipore, Billerica, MA, USA). Dilution and catalog number of each antibody were listed in Supplementary Table S1 in Supplementary Material. The secondary antibodies, sheep anti-rabbit or anti-mouse IgG-HRP, were from Thermo Fisher Scientific (Waltham, MA, USA). The proteins were visualized using an ECL detection kit (Thermo Fisher Scientific).

### Enzyme-Linked Immunosorbent Assay

BV2 cells were plated into 96-well plates and were pretreated with P7C3 for 2 h, and then exposed to LPS for 24 h. The production of these pro-inflammatory cytokines including PEG2, IL-6 and TNF-α in the culture media were detected using ELISA kits (BOSTER, Wuhan, China) according to the manufacturer’s instructions.

### Immunofluorescence Staining

BV2 cells were first pretreated with P7C3 for 2 h. Fifteen minutes after exposing to LPS, cells were fixed with 4% paraformaldehyde in PBS for 5 min, and then permeabilized with 0.25% Triton X-100 in PBS for 5 min. After washed with PBS for three times, cells were blocked with 0.5% fetal bovine serum. And the cells were then incubated with anti-p65 (Santa Cruz Biotechnology, Santa Cruz, CA, USA) for 6 h at room temperature, followed by an incubation with rhodamine-conjugated (red) secondary antibody for 2 h. Subsequently, the cells were stained with DAPI (Sigma, St Louis, MO, USA) for 5 min. Finally, the cells were observed using an inverted IX71 microscope system (Olympus, Tokyo, Japan).

### Subcellular Fractionation Assay

BV2 cells were first pretreated with P7C3 for 2 h. Fifteen minutes after exposing to LPS, cells were lysed in the fractionation buffer containing 3 mM CaCl2, 2 mM MgAc, 320 mM sucrose, 0.1 mM EDTA, 1 mM DTT, 0.5 mM phenylmethylsulfonyl fluoride (PMSF) and 0.5% NP-40 for 20 min on ice. After centrifugation for 15 min at 600 *g* at 4°C, the supernatants were collected as the cytoplasmic fraction. The pellets were washed twice with the fractionation buffer without NP-40 and was lysed in the nuclear lysis buffer containing 280 mM KCl, 0.2 mM EDTA, 1 mM DTT, 0.5 mM PMSF, 20 mM Hepes (pH 7.9), 25% glycerol, 1.5 mM MgCl2 and 0.3% NP-40 as the nuclear fraction.

### Immunohistochemistry

Male C57BL/6 mice, 25–30 g, were administrated with P7C3 or LPS described in the animal experiments above. After treatment, the mice were perfused with 0.9% saline followed by 4% paraformaldehyde in 0.1 M PBS (pH 7.4). The mice brains were then removed and post-fixed in the same fixation agent overnight at 4°C, followed by the treatment with the 30% sucrose at 4°C with another night. Serial 20 μM-thick mouse midbrain slices were cut with a freezing microtome. Immunohistochemical staining was conducted with anti-Iba1 antibody (Wako Chemicals, Tokyo, Japan), anti-GFAP and anti-TH antibodies (Millipore, Billerica, MA, USA) against six slices per mouse (120 μm interval). After incubation with primary antibodies at room temperature for 6 h, the slices were incubated with rhodamine (red)-or FITC (green)-conjugated secondary antibody (Invitrogen, Carlsbad, CA, USA) for 2 h. TH^+^ neurons were counted as described previously (Gu et al., 2017). Finally, the slices were stained with DAPI for 5 min and observed using an inverted IX71 microscope system (Olympus, Tokyo, Japan). Fluorescence intensity of Iba-1 and GFAP were anylized using ImageJ software (National Institutes of Health, Bethesda, MD, USA) as described previously (Guo et al., 2018).

### Luciferase Reporter Gene Assay

BV2 cells were transfected with Cignal lentiviral NF-κB reporter (QIAGEN, Hilden, Japan) according to the manufacturer’s recommendations for 24 h. Next, the culture media were substituted with fresh media and cultured for 24 h. Thereafter, the cells were cultured in the culture media containing with 2.5 μg/ml of puromycin for selection. Five days later, survived BV2 cells were selected and the stable BV2 cell line expressing NF-κB reporter construct was produced. Subsequently, the stable BV2 cells expressing NF-κB reporter constructs were treated with LPS in the presence or absence of P7C3. After treatment, the NF-κB promoter activity was detected with the luciferase assay kit (Promega, Madison, WI, USA) following the manufacturer’s recommendations.

### Nitrite Quantification

The nitric oxide (NO) level in each group with different treatment was detected using the Griess reagent (1% sulfanilamide, 2% phosphoric acid, 0.1% naphthylethylene). In brief, after the particular drug administration to the BV2 cells, 50 μL culture medium from each drug-treated BV2 cell group was collected and transferred into a 96-well plate. Subsequently, 50 μL Griess reagent was added into the collected culture media and incubated at room temperature for 10 min. The absorbance was read at 540 nm with a Microplate Reader (Multiskan MK3; Thermo Scientific).

### Statistical Analysis

The quantification analysis of immunoblots from three independent experiments was conducted using Photoshop 7.0 (Adobe, San Jose, CA, USA). Description of photoshop quantification of immunoblot bands were detailed in supplementary material. The data were analyzed using origin 6.0 (Originlab, Northampton, MA, USA). Significance of data between two groups was determined using a Mann and Whitney test for non-parametric data. Alternatively, Kruskal–Wallis analysis followed by a Conover-Iman test for multiple comparisons with Bonferroni correction between two independent groups, or a two-way ANOVA was applied. The criterion of significance was set at *P* < 0.05. The values are shown as the means ± SEM.

## Results

### P7C3 Inhibits LPS-Induced Production of Pro-inflammatory Factors in BV2 Cells

**Figure 1:**
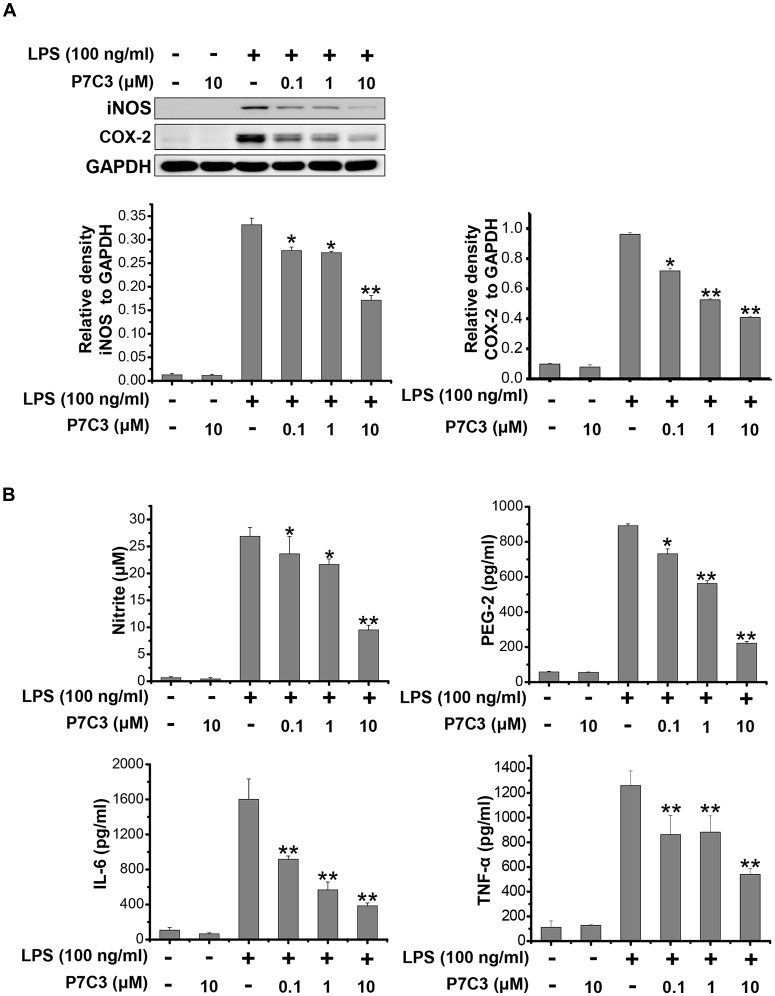
P7C3 inhibited lipopolysaccharide (LPS)-induced production of pro-inflammatory factors in BV2 cells. **(A)** BV2 cells were pretreated with P7C3 (0.1, 1, or 10 μM) for 2 h and then exposed to LPS (100 ng/mL) for 24 h. After treatment, the protein levels of iNOS, COX-2 and GAPDH were measured using immunoblot analysis. The quantification of the intensity of iNOS and COX-2 relative to GAPDH was shown in the two panels below. **(B)** BV2 cells were pretreated with P7C3 (0.1, 1, or 10 μM) for 2 h and then exposed to LPS (100 ng/mL) for 24 h. After culture, the cultured media were collected to measure the levels of nitrite using the Griess reagent, while the levels of PEG-2, IL-6 and TNFα were detected using ELISA assays. The values were presented as the means ± SEM from three independent experiments. Kruskal–Wallis test followed by the Iman-Conover method for multiple comparison between groups was performed, *P < 0.05, **P < 0.01 vs. the group treated with LPS alone.

It has been reported that LPS could significantly increase the production of many pro-inflammatory factors, including inducible NO synthase (iNOS), cyclooxygenase-2 (COX-2), interleukin-6 (IL-6) and TNF-α in BV2 microglial cell line (Hu et al., 2012; Gao et al., 2013). We wonder whether P7C3 has the potential to decrease the production of pro-inflammatory factors induced by LPS. In LPS-stimulated BV2 cells, a pretreatment of P7C3 markedly reduced the protein levels of iNOS and COX-2 in a dose-dependent manner without affecting the cell viability (Figure 1A, Supplementary Figure S1A). Furthermore, the LPS-induced release of pro-inflammatory cytokies including nitrite, prostaglandin E2 (PEG2), IL-6 and TNF-α in BV2 cell culture media were dramatically diminished by the pretreatment of P7C3 (Figure 1B).

### P7C3 Inhibits LPS-Induced Transcriptional Activation of Pro-inflammatory Factors in BV2 Cells

**Figure 2:**
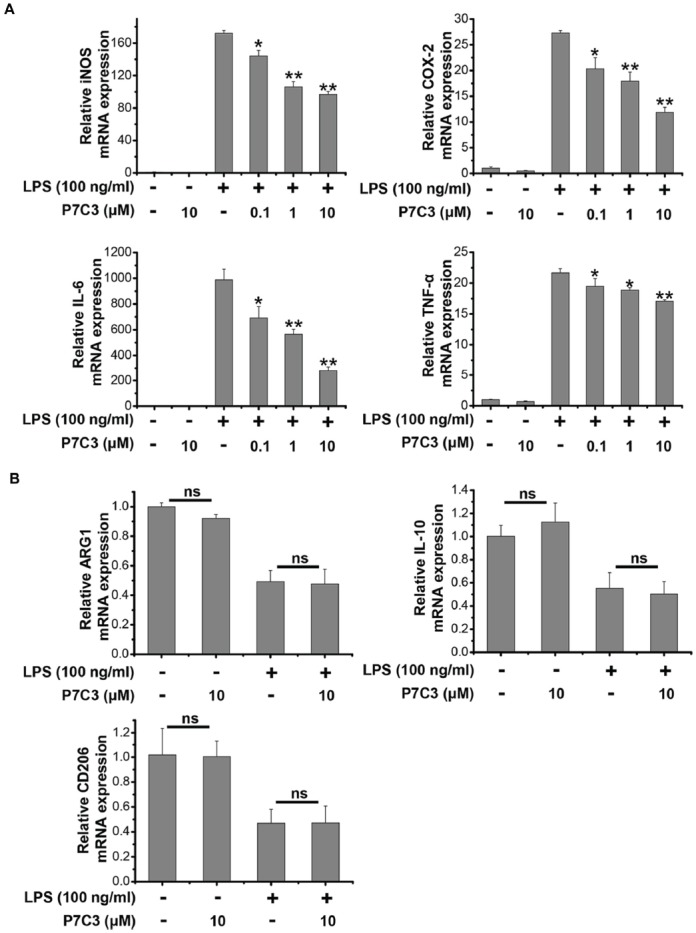

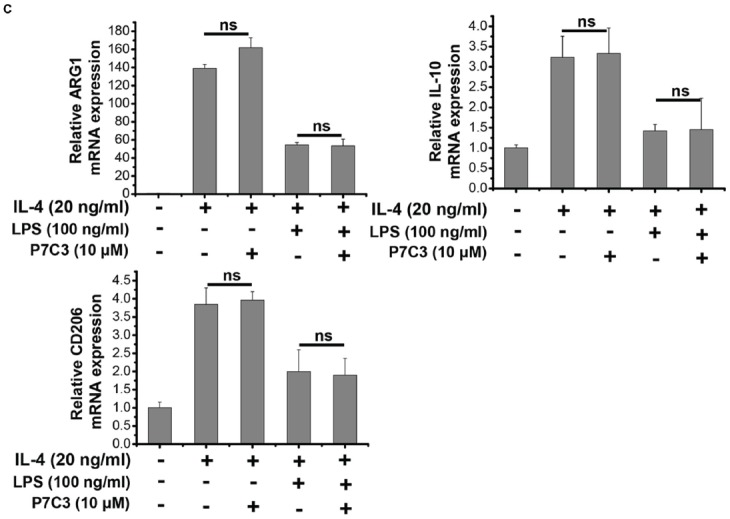
P7C3 inhibited LPS-induced transcriptional activation of pro-inflammatory factors in BV2 cells.** (A)** BV2 cells were pretreated with P7C3 (0.1, 1, or 10 μM) for 2 h and then exposed to LPS (100 ng/mL) for 6 h. After treatment, cell lysate was collected to measure the mRNA levels of *iNOS*, *COX-2*, *IL-6* and *TNFα* using qRT-PCR assays. The values are presented as the means ± SEM from three independent experiments. Kruskal–Wallis test followed by the Iman-Conover method for multiple comparison between groups was performed, *P < 0.05, **P < 0.01 vs. the group treated with LPS. **(B)** BV2 cells were pretreated with P7C3 (10 μM) for 2 h and then exposed to LPS (100 ng/mL) for 6 h. After culture, cell lysate was collected and the mRNA levels of *Arg1*, *IL-10*, *CD206* were measured using qRT-PCR assays. **(C)** BV2 cells were administrated with P7C3 (10 μM) for 2 h followed by IL-4 (20 ng/mL) treatment or IL-4 (20 ng/mL) plus LPS (100 ng/mL) treatment for 6 h. The mRNA levels of* Arg1*, *IL-10*, *CD206* in cell lysate were detected. The data from three independent experiments were presented as the means ± SEM.; Kruskal–Wallis test followed by the Iman-Conover method for multiple comparison between groups was performed. ns, not significantly different.

Since we showed that P7C3 was able to inhibit the production of pro-inflammatory factors in protein levels, we wonder whether LPS-induced pro-inflammatory factors could be transcriptionally repressed by P7C3. Stimulation of BV2 cells with LPS markedly increased mRNA levels of* iNOS*, *COX-2*, *IL-6* and *TNF-α*, however, P7C3 significantly reduced LPS-induced increases of mRNA levels of these pro-inflammatory factors (Figure 2A). It is widely accepted that microglia could be simply categorized into classical (M1) avtivation, expressing pro-inflammatory molecules mentioned above, and into alternative (M2) activation which can produce anti-inflammatory molecules like arignase1 (Arg1), interleukin-10 (IL-10) and CD206 (Jha et al., 2016). LPS is known as a representative M1 polarization inducer, while interleukin-4 (IL-4) induces M2 polarization (Cao and He, 2013). Sicne P7C3 inhibited M1 polarization, we wonder whether P7C3 could influence M2 polarization. In BV2 cells that were stimulated with or without the stimulation of IL-4, no effects of P7C3 on the expression of M2 microglial markers containing *Arg1*, *IL-10* and *CD206* were found (Figures 2B,C). Thus, our data suggest that P7C3 specifically suppresses LPS-induced M1 polarization of microglia.

### P7C3 Inhibits LPS-Induced Nuclear Translocation of NF-κB p65 Subunit in BV2 Cells

**Figure 3:**
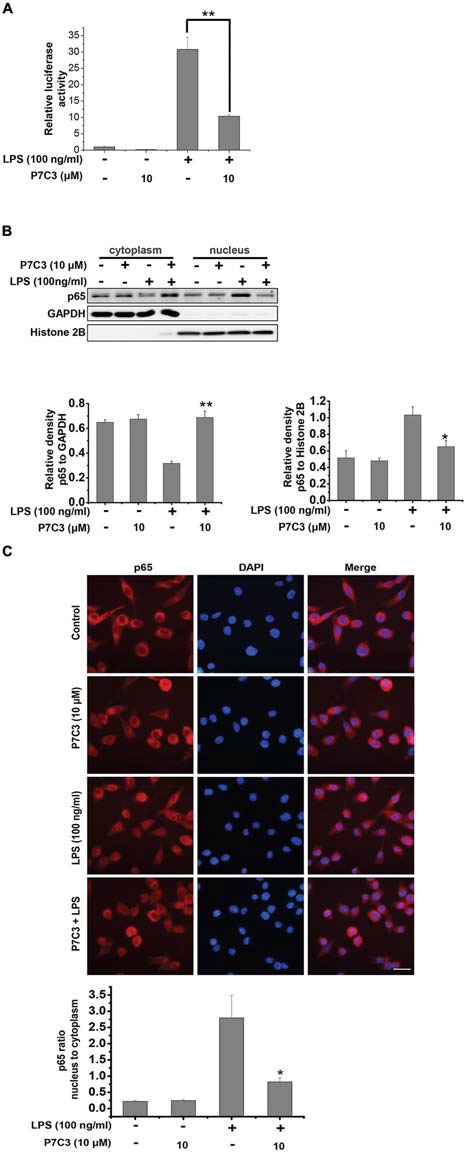
P7C3 inhibited LPS-induced nuclear translocation of NF-κB p65 subunit in BV2 cells.** (A)** The stable BV2 NF-κB reporter cells were pretreated with P7C3 (10 μM) for 2 h and then exposed to LPS (100 ng/mL) for 6 h. Then, the expressions of the NF-κB reporter gene were examined. Data from three independent experiments were presented as the means ± SEM. Mann and Whitney for comparison between groups, **P < 0.01.** (B)** BV2 cells were pretreated with P7C3 (10 μM) for 2 h and then exposed to LPS (100 ng/mL) for 15 min. After incubation, the cytoplasmic and nuclear fractions were separated using subcellular fractionation methods. The p65 protein levels in cytoplasm and nucleus were detected using immunoblot analysis. The quantitative analyses of the relative density of p65 to loading controls in cytoplasm (GAPDH) or in the nucleus (Histone 2B) were shown in the lower panels. The values were presented as the means ± SEM from three independent experiments. Mann and Whitney for comparison between groups, **P < 0.01 vs. the group treated with LPS. **(C)** BV2 cells that were treated as** (B)** were fixed and labeled with anti-p65 (red) antibodies. The nuclei were stained with DAPI (1 μg/mL; blue). The cells were then visualized using a fluorescent microscope. Scale bar, 10 μm. The ratio of nucleus to cytoplasm of p65 was quantified. The values are presented as the means ± SEM from three independent experiments. Kruskal–Wallis test followed by the Iman-Conover method for multiple comparison between groups was performed, *P < 0.05, vs. the group treated with LPS alone.

Since the transcription factor NF-κB plays an important role in transactivating pro-inflammatory gene expressions via its translocation to the nucleus upon LPS stimulation (Tak and Firestein, 2001), we wonder whether P7C3 exerts the anti-inflammatory effects through inhibiting NF-κB nuclear translocation in LPS-stimulated BV2 cells. We utilized a luciferase reporter gene assay to detect transcriptional activity of NF-κB in the BV2 cells that were stably expressing NF-κB reporter construct. In LPS-stimulated BV2 cells, the transcriptional activity of NF-κB was dramatically increased, whereas a pretreatment of P7C3 markedly decreased the reporter acitivity (Figure 3A). We further performed the nucleus-cytoplasm fractionation assay to detect the nuclear distribution of NF-κB p65 subunit. As expected, the nuclear NF-κB p65 subunit apparently increased in LPS-treated BV2 cells, while P7C3 significantly inhibited LPS-induced NF-κB nuclear translocation (Figure 3B). These findings were further supported by immunofluorescence staining, showing a decreased NF-κB nuclear translocation by P7C3 in LPS-stimulated BV2 cells (Figure 3C).

### P7C3 Suppresses LPS-Induced Degradation of IκBα by Inhibiting IKK Activation

**Figure 4:**
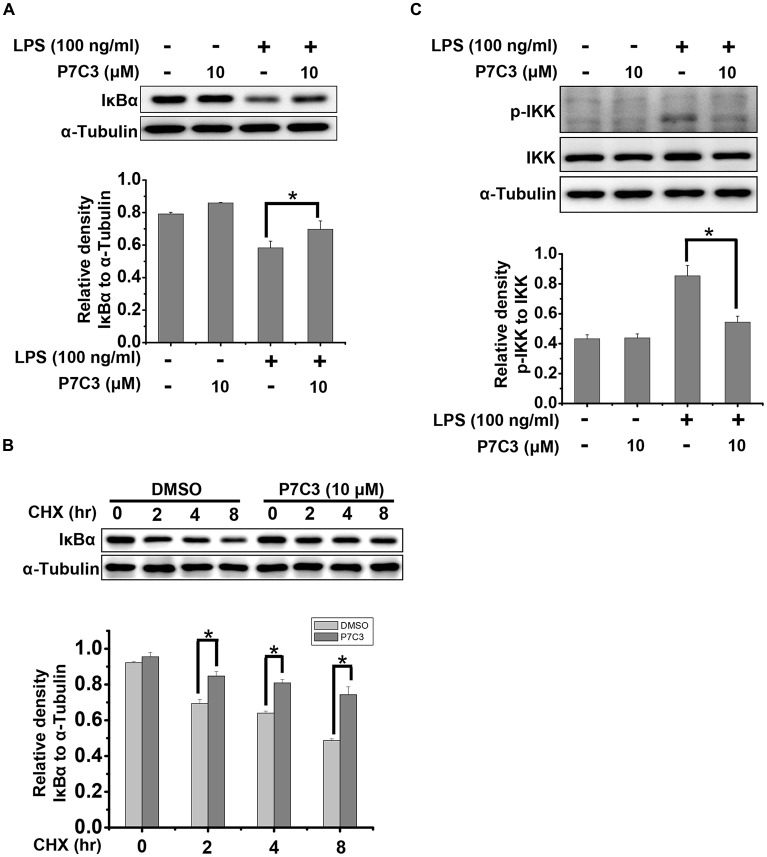
P7C3 suppressed LPS-induced degradation of inhibitory κB alpha (IκBα) by inhibiting IκB kinase (IKK) activation. **(A)** BV2 cells were treated with P7C3 (10 μM) for 2 h followed by the treatment with LPS (100 ng/mL) for 10 min. The IκBα and α-Tubulin protein levels were detected using immunoblot analysis. The bottom panel showed the band intensity of IκBα to α-Tubulin. The values were presented as the means ± SEM from three independent experiments. Mann and Whitney for comparison between groups, *P < 0.05. **(B)** BV2 cells were pretreated with P7C3 for 2 h and then exposed to cyclocheximide (CHX; 200 μg/mL) for the indicated time. The IκBα and α-Tubulin protein levels were detected using immunoblot analysis. The bottom panel showed the band intensity of IκBα to α-Tubulin. The values were presented as the means ± SEM from three independent experiments. Kruskal–Wallis test followed by the Iman-Conover method for multiple comparison between groups was performed, *P < 0.05. **(C)** BV2 cells were treated as **(A)**. The p-IKK, IKK and α-Tubulin protein levels were detected using immunoblot analysis. The bottom panel showed the band intensity of p-IKK to IKK. The values were presented as the means ± SEM from three independent experiments. Mann and Whitney for comparison between groups, *P < 0.05.

It is well known that IκBα is a crucial factor that interacts with and represses NF-κB nuclear tranlocation. Degradation of IκBα results in a release of NF-κB from the cytoplasm and a translocation into the nucleus (Beg and Baldwin, 1993). Therefore, we detected the degradation of IκBα triggered by LPS in the presence or absence of P7C3. P7C3 remarkably reduced the degradation of IκBα induced by LPS (Figure 4A). Moreover, Cyclocheximide (CHX) chase assay showed that P7C3 increased the stability of IκBα in BV2 cells that were subject to CHX, a protein synthesis inhibitor (Figure 4B). These data suggest that P7C3 inhibits the degradation of IκBα induced by LPS.

Degradation of IκBα depends on the ubiquitin-proteasome system. IκBα, upon phosphorylation, undergoes the proteasome-mediated degradation immediately (Skaug et al., 2009). IKK, which can be activated by phosphorylation, is capable of phosphorylating IκBα to promote IκBα degradation by the proteasome (Skaug et al., 2009). In LPS-stimulated BV2 cells, the phosphorylation of IKK was strikingly increased, whereas a pretreatment of P7C3 dramatically suppressed IKK phosphorylation (Figure 4C). These data provides evidence that P7C3 is able to repress IKK activation to inhibit the degradation of IκBα triggered by LPS.

### P7C3 Inhibits LPS-Induced Inflammation by Suppressing NF-κB Activation in Cultured Primary Microglia

**Figure 5:**
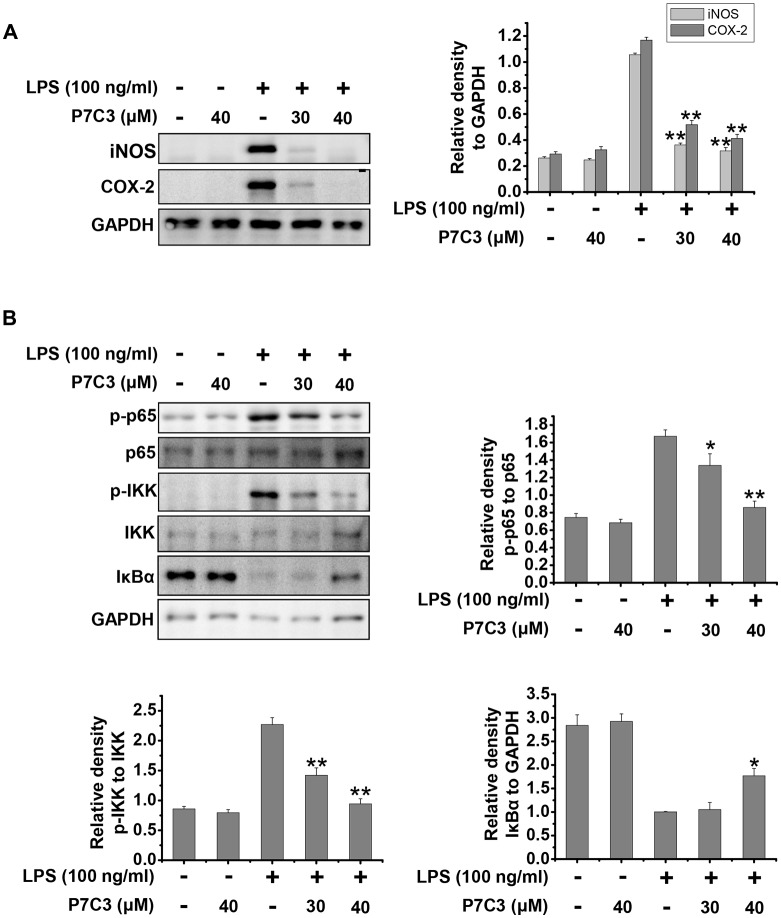
P7C3 inhibited LPS-induced inflammation by suppressing NF-κB activation in cultured primary microglia.** (A)** Cultured primary microglia were pretreated with various concentrations of P7C3 (30 or 40 μM) for 2 h followed by the treatment with LPS (100 ng/mL) for 24 h. After treatment, the protein levels of iNOS, COX-2 and GAPDH were measured using immunoblot analysis. The quantification of the intensity of iNOS and COX-2 relative to GAPDH was shown in the right panel. The values were presented as the means ± SEM from three independent experiments. Kruskal–Wallis test followed by the Iman-Conover method for multiple comparison between groups was performed, **P < 0.01 vs. the group treated with LPS alone. **(B)** Cultured primary microglia were pretreated with various concentrations of P7C3 (30 or 40 μM) for 2 h followed by the treatment with LPS (100 ng/mL) for 15 min. The p-p65, p65, p-IKK, IKK IκBα and GAPDH protein levels were detected using immunoblot analysis. The right panel showed the band intensity p-65 to p-65. The quantification of the intensity of p-IKK to IKK, or IκBα to GAPDH was showed in the lower panels. The values were presented as the means ± SEM from three independent experiments. Kruskal–Wallis test followed by the Iman-Conover method for multiple comparison between groups was performed, *P < 0.05, **P < 0.01 vs. the group treated with LPS alone.

Next, we utilized cultured primary microglia to further identify the effects of P7C3 on LPS-induced microglial activation. Similar to the results from BV2 cells, P7C3 significantly suppressed LPS-induced excessive expression of iNOS and COX-2 (Figure 5A). Meanwhile, P7C3 inhibited IKK or p65 phosphorylation, and blocked IκBα degradation (Figure 5B). These findings further confirmed the anti-inflammatory effects of P7C3 in LPS-stimulated microglia.

### P7C3 Suppresses Microglia-Mediated MES23.5 Cell Death

**Figure 6:**
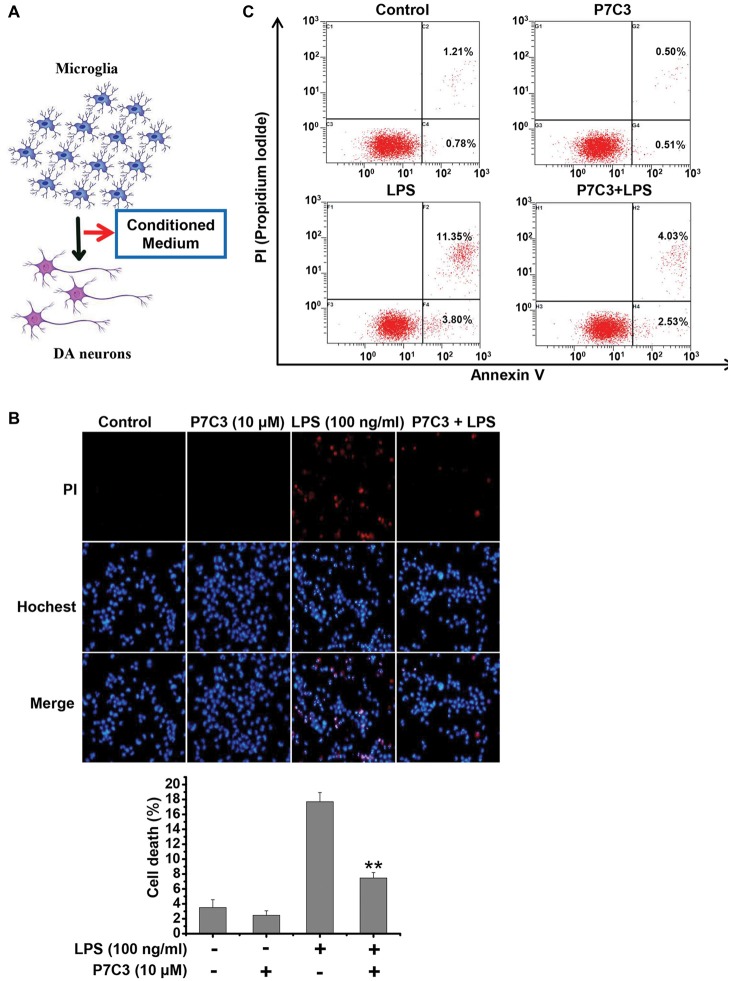
P7C3 suppressed microglia-mediated MES23.5 cell death. **(A)** Scheme of the conditioned medium (CM) assay using the media collected from LPS-stimulated BV2 cells with or without the pretreatment of P7C3 (10 μM) to culture the MES23.5 cells. **(B)** BV2 cells were treated with LPS (100 ng/mL) for 24 h with or without the pretreatment of P7C3 (10 μM), then the conditioned media from different groups were collected to culture the MES23.5 cells for 24 h. After treatment, the MES23.5 cells were subjected to Hoechst or propidium iodide (PI) staining to detect cell death. Scale bar, 10 μm. Cell death of MES23.5 cells was quantified. The values were presented as the means ± SEM from three independent experiments. Mann and Whitney for comparison between groups, **P < 0.01 vs. the groups with LPS-treated CM alone. **(C)** BV2 cells were treated as** (B)** and subjected to flow cytometry analysis.

It is widely accepted that activated microglia produce inflammatory molecules and damage neurons, including DA neurons (Block et al., 2007; Glass et al., 2010). Since we observed that P7C3 had the remarkable anti-inflammatory effects, we speculated that the neurotoxicity triggered by activated microglia may be alleviated by P7C3. To identify this possibility, we performed a conditioned medium (CM) assay (Figure 6A). CM collected from LPS-treated BV2 cells in the presence or absence of P7C3 were used to culture MES23.5 cells, a DA cell line. After above treatment, MES23.5 cells were subjected to Hochest and propidium iodide (PI) stainings, as well as the flow cytometry analysis for detecting cell apoptosis. The PI-positive cell number was significantly increased in MES23.5 cells that were cultured with CM from LPS-stimulated BV2 cells (Figure 6B). However, the PI-positive MES23.5 cells were dramatically decreased when the cells were cultured in CM from LPS-stimulated cells that were pretreated with P7C3 (Figure 6B). Moreover, the flow cytometry assay also showed that P7C3 decreased the toxicity of CM from LPS-stimulated BV2 cells (Figure 6C).

### P7C3 Inhibits Microglial Activation and Microglia-Mediated DA Neuronal Loss *in vivo*

**Figure 7:**
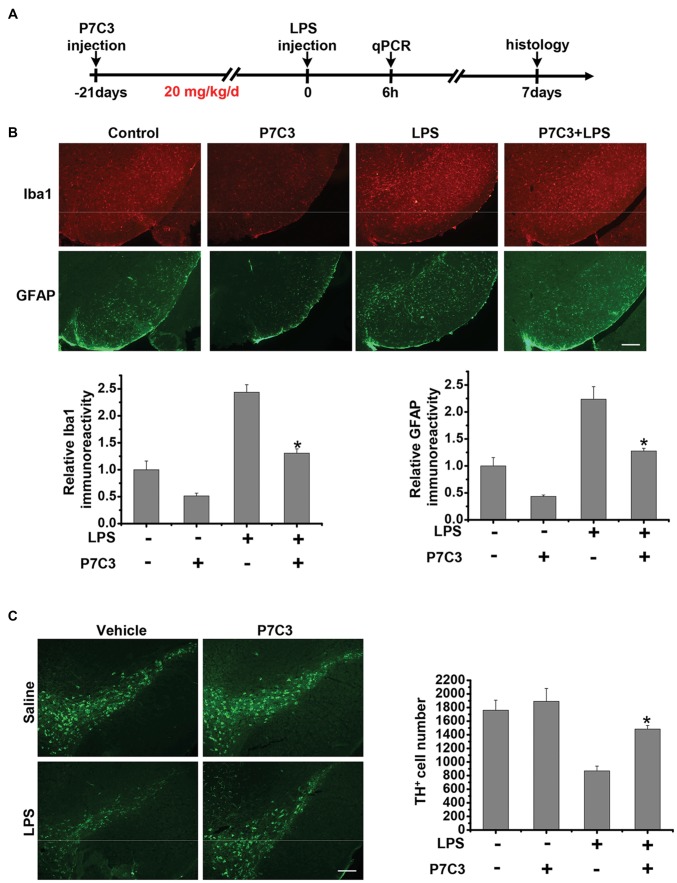

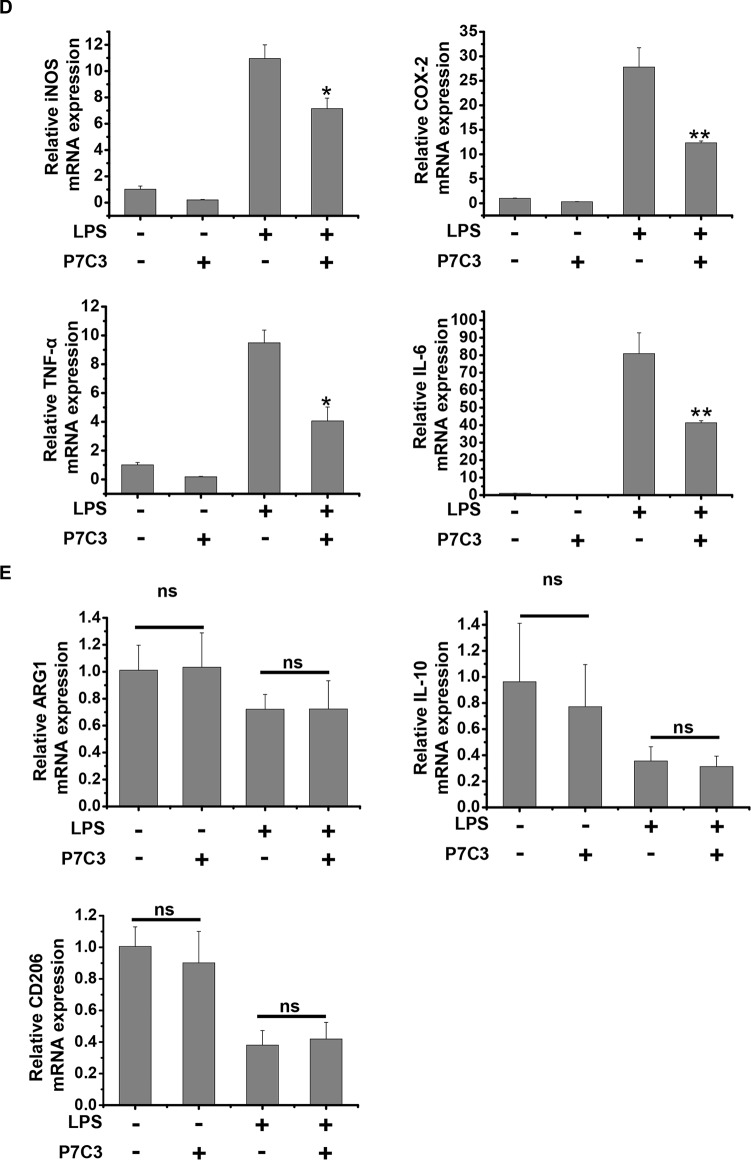
P7C3 inhibited microglial activation and microglia-mediated dopaminergic (DA) neuronal loss *in vivo*.** (A)** A schematic diagram of animal experimental procedure was shown. Seven days after the injection of LPS, mice were sacrificed and fixed with perfusion. The brains were post-fixed with fixing agent overnight at 4°C, followed by the treatment with 30% sucrose at 4°C for another one night. Serial 20 μM-thick mouse midbrain slices were cut using a freezing microtome. **(B)** Immunohistochemical staining was conducted using anti-Iba1 and anti-GFAP antibodies. Relative fluorescence intensity of Iba-1 and GFAP were shown in the lower panels. Scale bar, 100 μm, *n* = 6 per group. Mann and Whitney for comparison between groups, **P < 0.01 vs. the group treated with LPS alone. **(C)** Immunohistochemical staining was conducted using anti-tyrosine hydroxylase (TH) antibodies. The quantification of TH^+^ cell numbers was shown in the right panel. Scale bar, 100 μm, *n* = 6 per group. Mann and Whitney for comparison between groups, *P < 0.05 vs. the group treated with LPS alone using one-way ANOVA. **(D)** Six hours after the injection of LPS, mice midbrain were isolated and the total RNA was collected to measure the mRNA levels of *iNOS*, *COX-2*, *IL-6* and *TNFα* using qRT-PCR assays. *n* = 6 per group, Mann and Whitney for comparison between groups, *P < 0.05, **P < 0.01 vs. the group treated with LPS alone. **(E)** The total mRNA levels of *Arg-1*, *IL-10* and *CD206* from mice midbrains were also detected. *n* = 6 per group, Kruskal–Wallis test followed by the Iman-Conover method for multiple comparison between groups was performed, ns, not significantly different.

Since we have found that P7C3 had remarkable inhibitory effects on microglial activation *in vitro*, we wonder whether P7C3 has anti-inflammatory effects *in vivo*. Therefore, we established the mouse model of the excessive inflammatory response through LPS stereotactic injection into the mouse midbrain. Prior to LPS injection, a subsequent 21-days intraperitoneal injection of P7C3 was carried out to the mice (Figure 7A). Both Iba1 (a microglia marker) and GFAP (a astrocyte marker) were significantly increased in the SNpc after LPS administration (Figure 7B). However, in P7C3-administrated mice, LPS-induced expressions of Iba1 and GFAP in the SNpc were strikingly decreased (Figure 7B). Moreover, LPS-induced TH^+^ neuronal loss was greatly attenuated with the pretreatment of P7C3 in the SNpc (Figure 7C). To further testify the anti-inflammatory effects of P7C3 *in vivo*, we extracted total RNA from mouse midbrains of different treated groups. Consistent with the findings *in vitro*, the expressions of pro-inflammatory factors triggered by LPS including *iNOS*, *COX-2*, *IL-6* and *TNF-α* in mouse midbrains were remarkably decreased with the pretreatment of P7C3 (Figure 7D). Moreover, the expressions of anti-inflammatory factors were not changed in LPS-treated group either in the presence or absence of P7C3 (Figure 7E).

## Discussion

**Figure 8:**
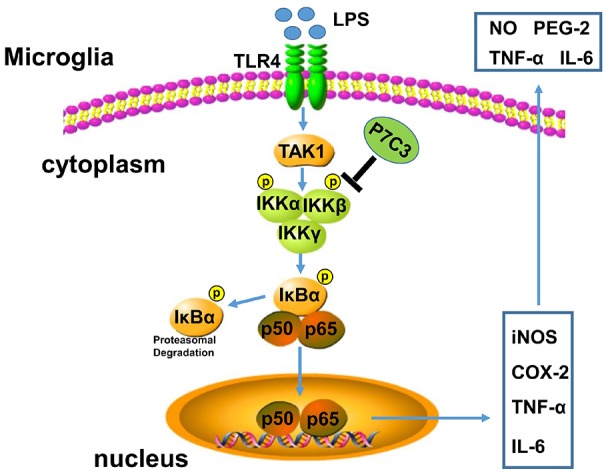
A schematic diagram shows the involvement of the IKK/IκBα/NF-κB pathway in P7C3-mediated repression of microglial activation. Once the Toll like receptor 4 (TLR4) is activated via its exogenous ligand LPS, TAK1 could be recruited to TLR4 and be autophosphorylated. Phosphorylation of TAK1 leads to an activation of the IKK complex containing IKK-α, IKK-β and IKK-γ. The IKK complex phosphorylates IκBα, leading to its ubiquitination and subsequent degradation, which allows NF-κB translocation to nucleus and targets inflammatory gene expressions including* iNOS*, *COX-2*, *TNF-α* and *IL-6*. However, a pretreatment of P7C3 inhibits IKK activation, thus, LPS-induced NF-κB activation is suppressed, leading a repression of inflammatory gene expressions.

P7C3 possesses powerful neuroprotective efficacy in neurodegenerative disease animal models (De Jesús-Cortés et al., 2012, 2015; Tesla et al., 2012; Voorhees et al., 2017). It has been reported that P7C3 protects DA neurons from neurotoxin-induced toxicity in PD animal models (De Jesús-Cortés et al., 2012, 2015; Gu et al., 2017). It is well known that inflammation triggered by microglia plays an important role in promoting DA neurons loss through motivating pro-inflammatory factor expressions, thereby accelerating the progression of PD (Liu and Bing, 2011; Kempuraj et al., 2016). Here, we demonstrate that P7C3 has roles in anti-inflammation. P7C3 inhibits LPS-induced activation of microglia by targeting the NF-κB pathway, which decreases LPS-induced production of inflammatory factors to protect DA neurons *in vivo* and *in vitro* (Figure 8).

LPS-induced inflammatory neurotoxicity depends mainly on the excessive production of pro-inflammatory factors including iNOS, COX-2, IL-6, TNF-α by microglia (Okun et al., 2009; Liu and Bing, 2011). The inhibitors of iNOS and COX-2 have neuroprotective effects by suppressing microglial activation and inflammatory damage (Li et al., 2003; Pawlak et al., 2012; Xia et al., 2015). TNFα and IL-6 play central roles in initiating and regulating the cytokine cascade in inflammatory response (Brown and Neher, 2010). Microglia could be categorized into M1 and M2 phases, although this classification seems to be oversimple (Jha et al., 2016). M1 polarization makes microglia detrimental with exaggerated pro-inflammatory factors, while M2 polarization of microglia produces more anti-inflammatory mediators like Arg1, IL-10 and CD206 to protect neurons from inflammatory damage (Nakagawa and Chiba, 2014; Jha et al., 2016). In our observations, P7C3 inhibits the production of pro-inflammatory factors upon LPS stimulation, but not influences the production of anti-inflammatory mediators both *in vivo* and *in vitro*, suggesting that P7C3 might function specifically in regulating M1 polarization.

Two common transcription factors NF-κB and AP-1 are both involved in LPS-induced microglial activation (Moriyama et al., 2006; Park et al., 2010). NF-κB activation relies on the nuclear translocation of NF-κB p65 (Brasier, 2006), while AP-1 activation depends on the phosphorylation and activation of c-Jun and c-Fos (Hess et al., 2004; Zenz et al., 2008). LPS stimulation in microglia could remarkably enhance the transcriptional activity of NF-κB and AP-1 simultaneously (Moriyama et al., 2006). Under normal conditions, IκBα binds to dimerized NF-κB in the cytoplasm and represses nuclear translocation of NF-κB (Brasier, 2006). However, IκBα can be rapidly phosphorylated and polyubiquitinated upon LPS stimulation, leading to a degradation by the proteasome, which causes the release of NF-κB from IκBα and a translocation into nucleus. (Brasier, 2006). In our observations, IκBα degradation and NF-κB nuclear translocation were significantly suppressed with the pretreatment of P7C3 in LPS-treated BV2 cells and cultured primary microglia. However, LPS-induced phosphorylation of c-Jun and c-Fos was not changed obviously by the pretreatment of P7C3 (Supplementary Figure S1B). Meanwhile, LPS-induced phosphorylation of p65 and IKK can be blocked by P7C3, further suggesting the involvement of NF-κB in the anti-inflammatory effects by P7C3.

The activation of TLR4 on the cell membrane by LPS activates NF-κB via the MyD88-IRAK-TRAF6-TAK1 signaling complex (Lee and Lee, 2002; Schmalz et al., 2011; Madera-Salcedo et al., 2013). TAK1 is recruited to TLR4 upon LPS stimulation, and dissociates from the receptor presumably to bifurcate the signal into two main branches, the IKK-dependent cascade and the MAPK signaling pathway (Madera-Salcedo et al., 2013). In brief, TLR4 activation results in the activation of a signalosome consisting of TRAF6, IRAK1 and TAK1, and subsequent autophosphorylation of TAK1, leading to an activation of the IKK complex consisting of IKK-α, IKK-β and IKK-γ. The IKK complex phosphorylates IκBα, leading to its ubiquitination for degradation, which allows NF-kB translocation to nucleus to transactivate its target gene expressions (Gatheral et al., 2012; Kim et al., 2013). TAK1 also phosphorylates MAPKs, leading to AP-1 activation and gene transcriptions (Karin, 1996; Mihaly et al., 2014). In our study, P7C3 did not inhibit LPS-induced MAPK activation and TAK1 phosphorylation (Supplementary Figures S2A,B), but largely suppresses IKK activation. The prediction for interactions showed that there exists one hydrogen bond between P7C3 and IKK, although the affinity of P7C3 with IKK was weaker than that of the IKK inhibitor IMD0354 (Supplementary Figure S3), suggesting that IKK might be a target of P7C3. Neuroinflammation, which is characterized by microglial activation that may be induced by genetic or environmental factors, is tightly associated with PD processes (Glass et al., 2010; Yan et al., 2014). In the present study, we observed that P7C3 decreases the toxicity of CM from LPS-stimulated microglia in cultured BV2 cells. Moreover, in LPS-administrated mice, P7C3 also exhibits anti-inflammatory effects, which inhibits the production of pro-inflammatory factors and protects DA neurons against LPS-induced neuronal loss.

In summary, we show that P7C3 inhibits IKK activation to suppress LPS-induced microglial activation, protecting DA neurons from inflammation-induced damage *in vivo* and *in vitro*. Thus, our study provides evidence that P7C3 plays roles in anti-inflammation via NF-κB pathways.

## Author Contributions

QH, JW, CG and GW designed the study. CG analyzed the data and drafted the manuscript. GW revised the manuscript. QH, JW and CG performed most of the experiments. HR and C-FL discussed the data. CM contributed critical reagents. HR analyzed the data.

## Conflict of Interest Statement

The authors declare that the research was conducted in the absence of any commercial or financial relationships that could be construed as a potential conflict of interest.
