# Supplementary material for: P7C3 Inhibits LPS-Induced Microglial Activation to Protect Dopaminergic Neurons Against Inflammatory Factor-Induced Cell Death in vitro and in vivo
Source: Front Cell Neurosci. 2018 Nov 5;12:400. doi: 10.3389/fncel.2018.00400 (PMC6230654; doi:10.3389/fncel.2018.00400)
Supplement: Supplementary file 1 [file Data_Sheet_1.PDF]

Supplementary material

P7C3 inhibits LPS-induced microglial activation to protect dopaminergic neurons against inflammatory factor-induced cell death *in vitro* and *in vivo*

Chao Gu[1,†], Qingsong Hu[1,†], Jiayuan Wu[2,†], Chenchen Mu[1], Haigang Ren[1], Chun-Feng Liu[3,4] and Guanghui Wang[1*]

***Correspondence:**

Corresponding author: Guanghui Wang

Tel: 86-512-65884845, Fax: 86-512-65884845; Email: wanggh@suda.edu.cn

Supplementary Figure 1

A

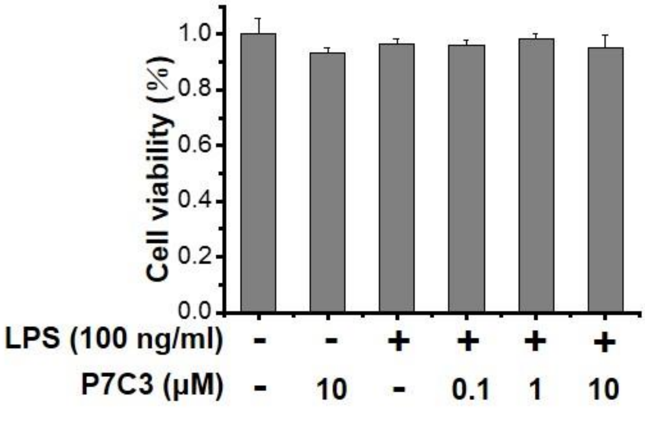

B

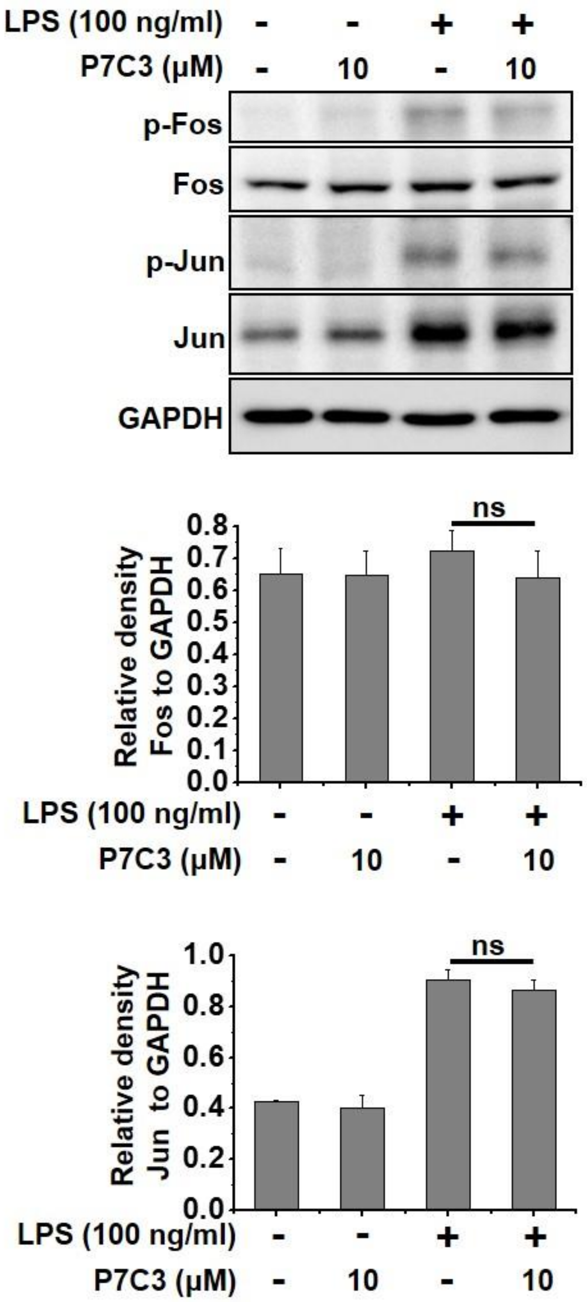

Supplementary Figure 1. (A) BV2 cells were pretreated with P7C3 (0.1, 1, or 10 μM) for 2 h and then exposed to LPS (100 ng/mL) for 24 h. The normalized BV2 cells viability in different groups was measured using MTT assay. No significant changes were observed in any group. **(B)** BV2 cells were treated with P7C3 (10 μM) for 2 h followed by the treatment with LPS (100 ng/mL) for 2 h. The protein levels of p-Fos,

Fos, p-Jun, Jun and GAPDH were detected with immunoblot analysis. The lower two panels showed the band intensity Fos and Jun to GAPDH. The data from three independent experiments are presented as the means ± S.E.M. Kruskal–Wallis test followed by the Iman-Conover method for multiple comparison between groups was performed, ns, not significantly different.

Supplementary Figure 2

A

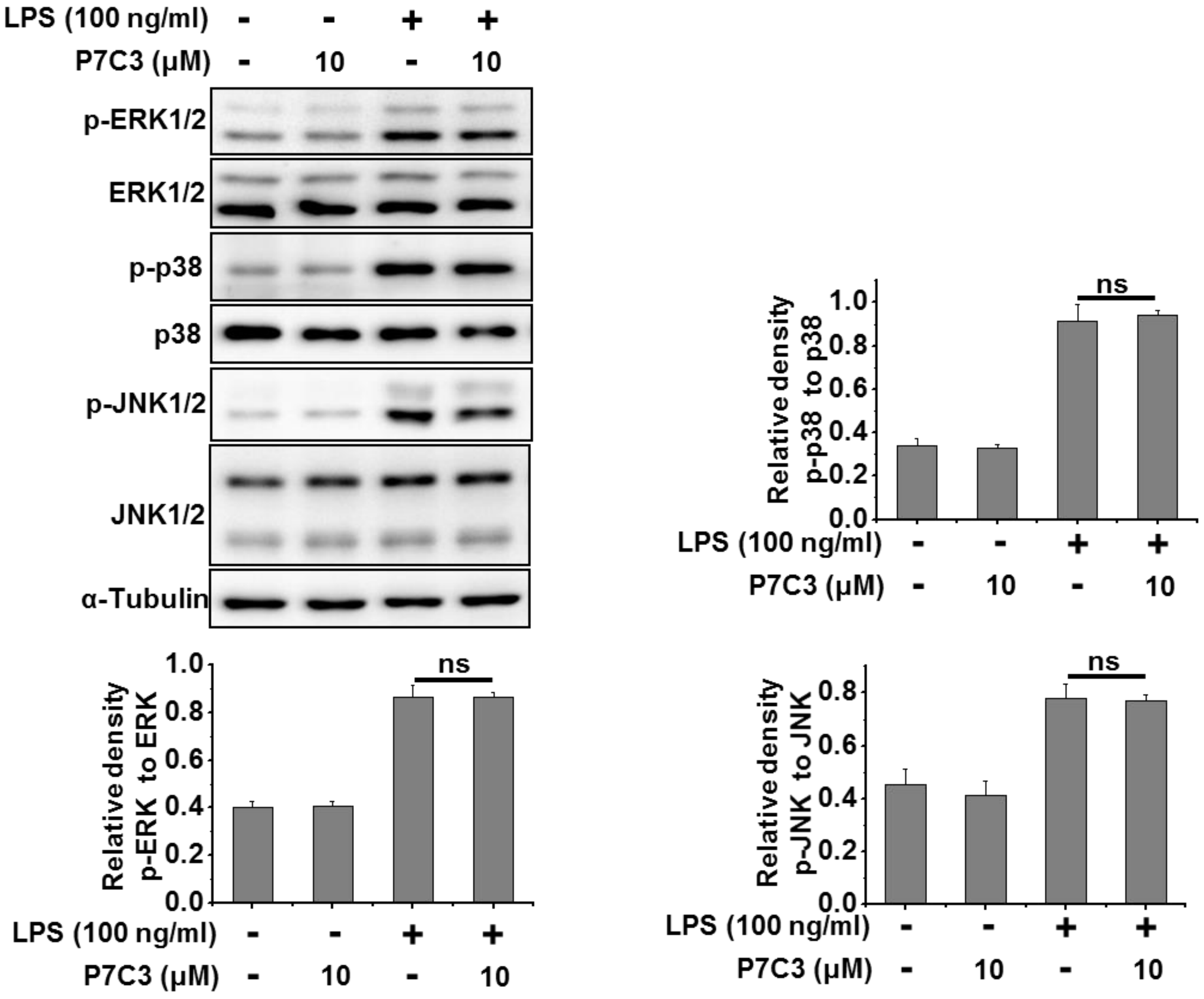

B

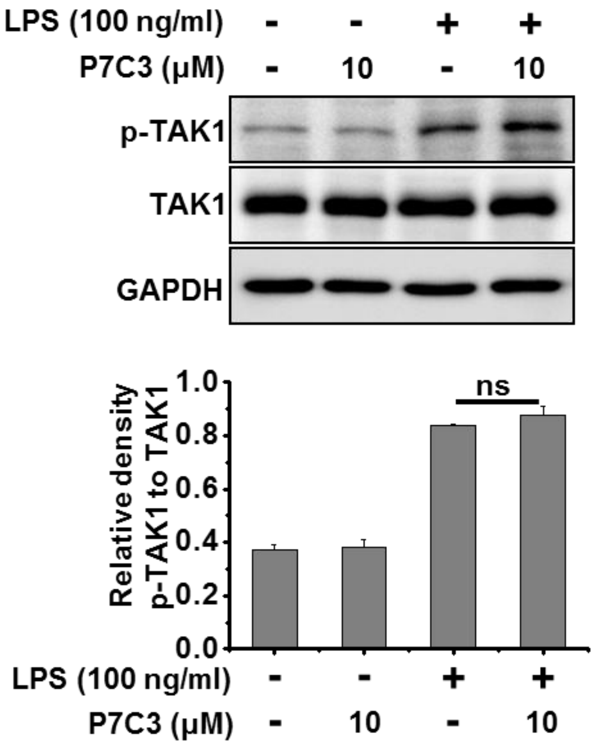

Supplementary Figure 2. (A) BV2 cells were administrated with P7C3 (10 μM) for 2 h followed by treatment with LPS (100 ng/mL) for 10 min. The protein levels of p-ERK1/2, ERK, p-p38, p38, p-JNK1/2, JNK1/2 and α-Tubulin were detected with immunoblot analysis. The surrounding panels showed the band intensity p-ERK1/2 to ERK1/2, p-p38 to p38 and p-JNK1/2 to JNK1/2. The data from three independent experiments are presented as the means ± S.E.M. Kruskal–Wallis test followed by the Iman-Conover method for multiple comparison between groups was performed, ns, not significantly different. **(B)** BV2 cells were administrated with P7C3 (10 μM) for 2 h followed by treatment with LPS (100 ng/mL) for 10 min. The protein levels of p-TAK1, TAK1 and GAPDH were detected with immunoblot analysis. The bottom panels showed the band intensity p-TAK1 to TAK1. The data from three independent experiments are presented as the means ± S.E.M. Kruskal–Wallis test followed by the Iman-Conover method for multiple comparison between groups was performed. ns, not

significantly different.

Supplementary Figure 3.

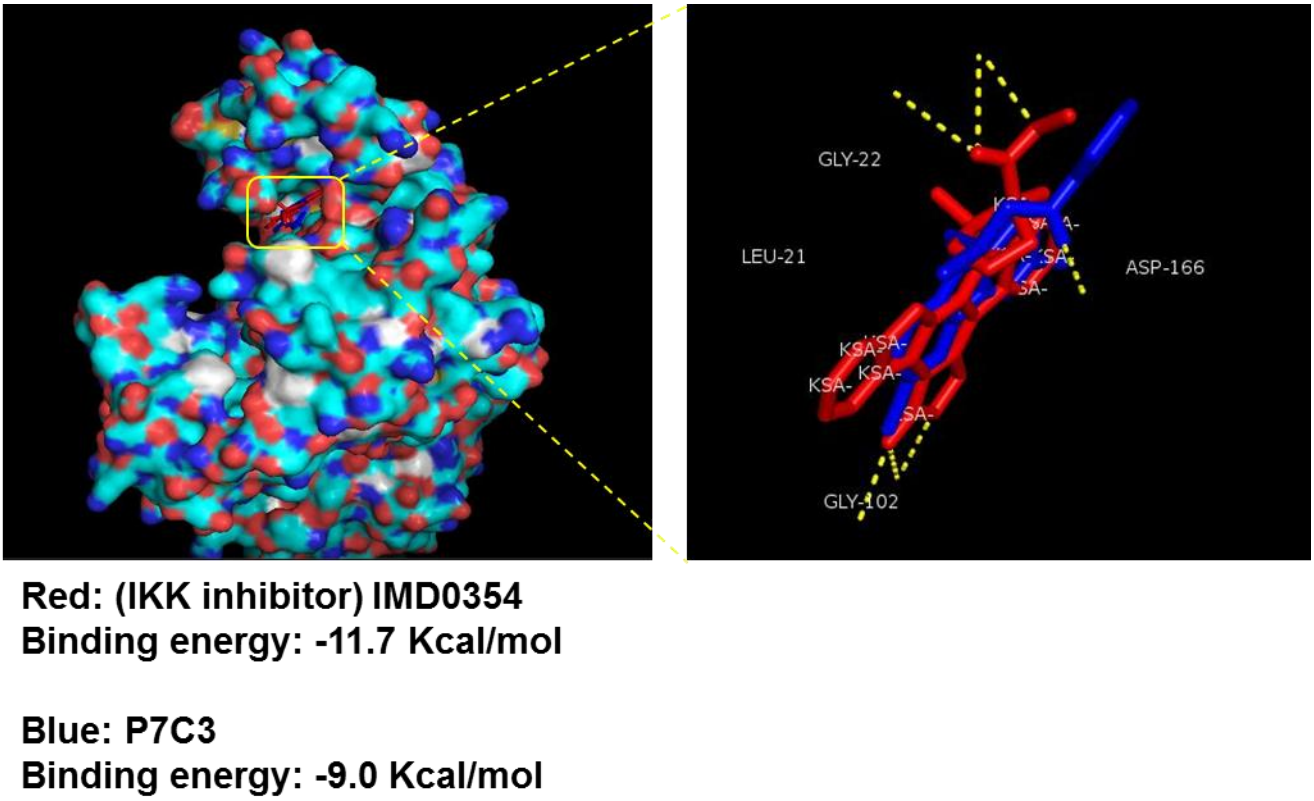

Supplementary Table 1. Concentrations of every antibodies and catalogue numbers of each of them.

Antibody	Concentration	Catalogue number
α-Tubulin	1:10000	ab7291
COX-2	1:2500	ab15191
Histone 2B	1:2000	ab1790
IκBα	1:2000	ab32518
iNOS	1:2000	ab15323
p-p65	1:1000	3033S
IKK	1:1000	61294S

p-IKK	1:500	2697S
p65	1:500	SC-71675
GAPDH	1:5000	CB1001
Iba1	1:1000	019-19741
GFAP	1:1000	MAB360
TH	1:1000	MAB152

Detailed description of photoshop quantification of western blot bands

1. Open the scanned image in Photoshop.

2. Under Image>Mode, check the grayscale option if it's not already selected.

3. Under Image>Adjustments, select Invert (or press Ctrl+I). Now the dark parts of the film are light, and the light parts are dark.

4. Choose the lasso tool from the tool palette.

5. On the first band, use the lasso tool to draw a line all the way around the edges of the first band.

6 .Go to Image>Histogram to display the histogram for the current selection.

7. The histogram information includes a "Mean" value and a "Pixels" value. Record these two numbers for your selection. The Mean value is the average gray value (from 0 to 255) for the area inside your selection. The Pixels value is the number of pixels contained in your selection area.

8. On your picture, use the lasso tool to draw around the next band. Record the Mean and Pixel values for this selection. Repeat for the rest of your bands.
